# IL-27 Regulated CD4^+^IL-10^+^ T Cells in Experimental Sjögren Syndrome

**DOI:** 10.3389/fimmu.2020.01699

**Published:** 2020-08-11

**Authors:** Jingjing Qi, Zhuoya Zhang, Xiaojun Tang, Wenchao Li, Weiwei Chen, Genhong Yao

**Affiliations:** ^1^Department of Rheumatology and Immunology, Nanjing Drum Tower Hospital, The Affiliated Hospital of Nanjing University Medical School, Nanjing, China; ^2^Department of Immunology, College of Basic Medical Science, Dalian Medical University, Dalian, Liaoning, China

**Keywords:** Sjögren syndrome, CD4^+^IL-10^+^ T cells, interleukin-27, interleukin-10, immunosuppression

## Abstract

Interleukin 27 (IL-27) plays diverse immune regulatory roles in autoimmune disorders and promotes the generation of IL-10–producing CD4^+^ T cells characterized by producing the immunosuppressive cytokine IL-10. However, whether IL-27 participates in pathological progress of Sjögren syndrome (SS) through regulating CD4^+^IL-10^+^ T cells remains unknown. Here we aimed to explore the potential role of IL-27 and CD4^+^IL-10^+^ T cells in the pathogenesis of SS. The IL-27 gene knockout non-obese diabetic (*Il-27*^−/−^NOD) mice were generated and injected with exogenous IL-27. Exogenous injection of IL-27 and neutralization of IL-27 with anti–IL-27 antibody in NOD mice were performed. The histopathologic changes in submandibular glands, lacrimal glands and lung, salivary flow rate, and percentages of CD4^+^IL-10^+^ T cells were determined. And, ovalbumin-immunized C57L/B6 mice were injected with IL-27 to detect the percentage of CD4^+^IL-10^+^ T cells. *In vitro*, splenic naive T cells from C57L/B6 mice were cultured with IL-27 for 4 days to induce the differentiation of CD4^+^IL-10^+^ T cells. In addition, IL-27, IL-10, and CD4^+^IL-10^+^ T cells were determined in health control and SS patients. The results showed that *Il-27*^−/−^NOD mice had more severe disease and lower level of CD4^+^IL-10^+^ T cells than control mice. And IL-27 promoted the generation and differentiation of CD4^+^IL-10^+^ T cells *in vivo* and *in vitro* significantly. In agreement with the findings in the SS-like mice, patients with SS showed lower levels of IL-27, IL-10, and CD4^+^IL-10^+^ T cells. Our findings indicated that IL-27 deficiency aggravated SS by regulating CD4^+^IL-10^+^ T cells. Targeting IL-27 and CD4^+^IL-10^+^ T cells may be a novel therapy for patients with SS.

## Introduction

Sjögren syndrome (SS) is a chronic autoimmune disease that typically presents with dry eyes and mouth as a result of lymphocytic infiltration in salivary (SGs) and lacrimal glands (LGs). Sjögren syndrome is characterized by the presence of autoreactive T and B cells in exocrine glands and circulating antibodies against several autologous antigens, such as the autoantibodies against SS antigen A (SSA)/Ro and SS antigen B (SSB)/La and the Fc fragment of immunoglobulin G (1, 2). The current treatments of SS are limited to be symptomatic, as a result of the elusive pathogenesis and diverse syndrome. According to reports, the dysregulated cytokine network contributes to the occurrence and development of SS. Thus, targeting to cytokine as the potential therapies in SS should be explored (3).

Interleukin 27 (IL-27) is a heterodimeric immunological factor of the IL-12 cytokine family and consists of p28 and Epstein-Barr virus–induced gene 3 (EBi3) subunits. Interleukin 27 mainly produced by antigen-presenting cells (APCs) upon stimulation of innate immune receptors (4–7). The IL-27 receptor is composed of the unique chain IL-27Rα (also called TCCR or WSX-1) and gp130 (8). Interleukin 27Rα is widely expressed in the immune cells (9). The ligation of IL-27 and its receptor induce intracellular signaling via heterogeneous Jak/STAT pathways, with predominant activation of STAT1 and STAT3 (10–12). Interleukin 27 was preliminarily characterized as a pro-inflammatory cytokine with T_H_1 induction. However, studies with infectious and autoimmune inflammatory models had reported that IL-27Rα deficiency mice developed excessive, pathological inflammation responding to a variety of challenges (13). And the anti-inflammatory role of IL-27 signaling has been illustrated in many recent studies (6, 14–17).

Hunter and colleagues showed the molecular mechanisms underlying suppressive characters of IL-27, which indicated that IL-27 could reduce IL-2 production during T_H_1 differentiation and promote the development of regulatory T (Treg) cells specialized to control T_H_1-mediated immunity at local sites of inflammation (18, 19). Interleukin 27 signaling in DCs played a key role in antigen-induced peripheral tolerance, which relied on the ability of IL-27 to induce T cell–derived IL-10 and interferon γ (IFN-γ) (20). And the study found that IL-27 and IL-6 induced T_H_1 and T_H_2 cells, as well as T_H_17 cells to secrete IL-10 (21). Interleukin 27 promoted the expression of inhibitory receptors on T cells *in vivo* and *in vitro* (22). Furthermore, studies reported that IL-27 drove the generation (23) and differentiation of IL-10–producing murine type 1 regulatory T (Tr1) cells by inducing three key factors: the transcription factor c-Maf, the cytokine IL-21, and the costimulatory receptor ICOS (24, 25).

Tr1 cells are a subset of T cells that have strong immunosuppressive properties and predominantly produce IL-10 with variable amounts of IFN-γ, IL-2, and transforming growth factor β (TGF-β), but do not express transcription factor Fork head box 3 (Foxp3) (23, 26, 27). There is a lot of research focused on Tr1 cells to suppress innate and adaptive immunity to alleviate inflammatory pathologies, in particular autoimmune diseases (28–31). Interleukin 27 could limit autoimmune diseases by stimulating IL-10–secreting T cells (6, 16, 32). Nevertheless, the role of IL-27 and CD4^+^IL-10^+^ T cells in SS remained unknown.

Here, our results showed that CD4^+^IL-10^+^ T cells related to SS pathogenesis and reduced generation of CD4^+^IL-10^+^ T cells was ascribed to decreased IL-27 in SS. Our findings indicated that targeting to IL-27 and CD4^+^IL-10^+^ T cells is a new direction for the SS treatment.

## Materials and Methods

### Study Population

A total of 31 SS patients and 34 health controls (HCs) from the Department of Rheumatology and Immunology, The Affiliated Drum Tower Hospital of Nanjing University Medical School, Nanjing University, were enrolled. Written informed consent was obtained from all subjects. Whole-blood samples were obtained. Plasma was isolated and frozen at −80°C until use. The study was approved by the ethics committee of our institute.

### Mice

Seven-week-old female NOD and IL-27 gene knockout female NOD (*Il-27*^−/−^NOD) and 5-week-old female C57L/B6 mice were purchased from the Model Animal Research Center of Nanjing University and maintained under specific-pathogen–free conditions in the animal center of the Affiliated Drum Tower Hospital of Nanjing University Medical School.

### Salivary Flow Rate

After anesthetization, the mice were stimulated with pilocarpine 0.1 mg pilocarpine/kg body weight injected intraperitoneally (i.p.). The whole saliva was obtained from the oral cavity for 15 min. Saliva volume was determined gravimetrically.

### Histological Analysis

For histological analysis, mice were euthanized. Submandibular glands, LGs, and lung tissues were collected and embedded in paraffin for hematoxylin and eosin staining.

### Preparation of Single Cell Suspensions

Spleen was isolated, and the red blood cells were lysed with lysing buffer for single cell suspension preparation. Peripheral blood mononuclear cells in blood from SS patients and healthy volunteers were isolated with Ficoll-Hypaque by density gradient centrifugation.

### Flow Cytometry

Antibodies (eBioscience) were diluted at optimal concentration for cell immunostaining. To avoid non-specific binding to Fc receptors, isotype-matched antibodies were used as controls. For analysis of intracellular IL-10, cells were stimulated with 20 ng/mL PMA plus 1 μg/mL ionomycin with 5 μg/mL of brefeldin A (Enzo LifeScience, East Farmingdale, NY, USA) at 37°C for 4 h before harvest. First, surface CD4 with anti–mouse/human CD4–fluorescein isothiocyanate was stained. After that, cells were fixed with Cytofix/Cytoperm solution (BD Pharmingen), incubated with anti–mouse IL-10–APC or anti–human IL-10–APC and analyzed on a FACS Calibur flow cytometer (BD Biosciences, Mountain View, CA, USA).

### Immunization

Seven-week-old female C57L/B6 mice were sensitized with 10 μg ovalbumin (OVA) in complete Freund's adjuvant intradermally as the described methods (33).

### *In vivo* Treatment With IL-27 and Anti–IL-27

In the IL-27 treatment experiments, 11-week-old female NOD mice or OVA-immunized 8-week-old female C57L/B6 mice were injected with 200 ng/mouse IL-27 (BioLegend) once a day for a total of seven times. The control mice were injected with same volume of phosphate-buffered saline (PBS). In the anti-IL-27 treatment experiments, female 11-week-old NOD mice were injected with 0.5 mg/mouse anti-IL-27 (BioLegend) or with commensurable IgG2a isotype once i.p. Mice were sacrificed on the seventh day.

### *In vitro* Tr1 Cell Differentiation

Splenic CD4^+^CD62L^+^ naive T cells were purified with Micro Beads (Miltenyi Biotec) from 6- to 8-week-old female C57L/B6 mice, with purity of more than 90%. Then naive T cells are cultured in a 96-well plate bound with 4 μg/mL anti-CD3e antibodies (eBioscience) at a density of 1 × 10^6^/mL in RPMI 1640 supplemented with 10% fetal bovine serum and 100 U/mL penicillin/streptomycin, in the presence of 2 μg/mL anti-CD28 antibodies with 20 ng/mL mouse recombinant IL-27 (rIL-27) (BioLegend) or not for 4 days.

### Cytokine Quantification

The plasma IL-10 and IL-27 were measured by standard sandwich enzyme-linked immunosorbent assay (ELISA) kits (R&D Systems) according to the manufacturer's instructions.

### Statistical Analysis

Statistical analysis was performed with Prism software version 5.0 (GraphPad Software). Differences in means ± SEM were evaluated with Student *t* test or one-way analysis of variance followed by Dunnett test. *P* < 0.05 was considered statistically significant.

## Results

### *Il-27*^−/−^NOD Mice Displayed Severe SS

**Figure 1:**
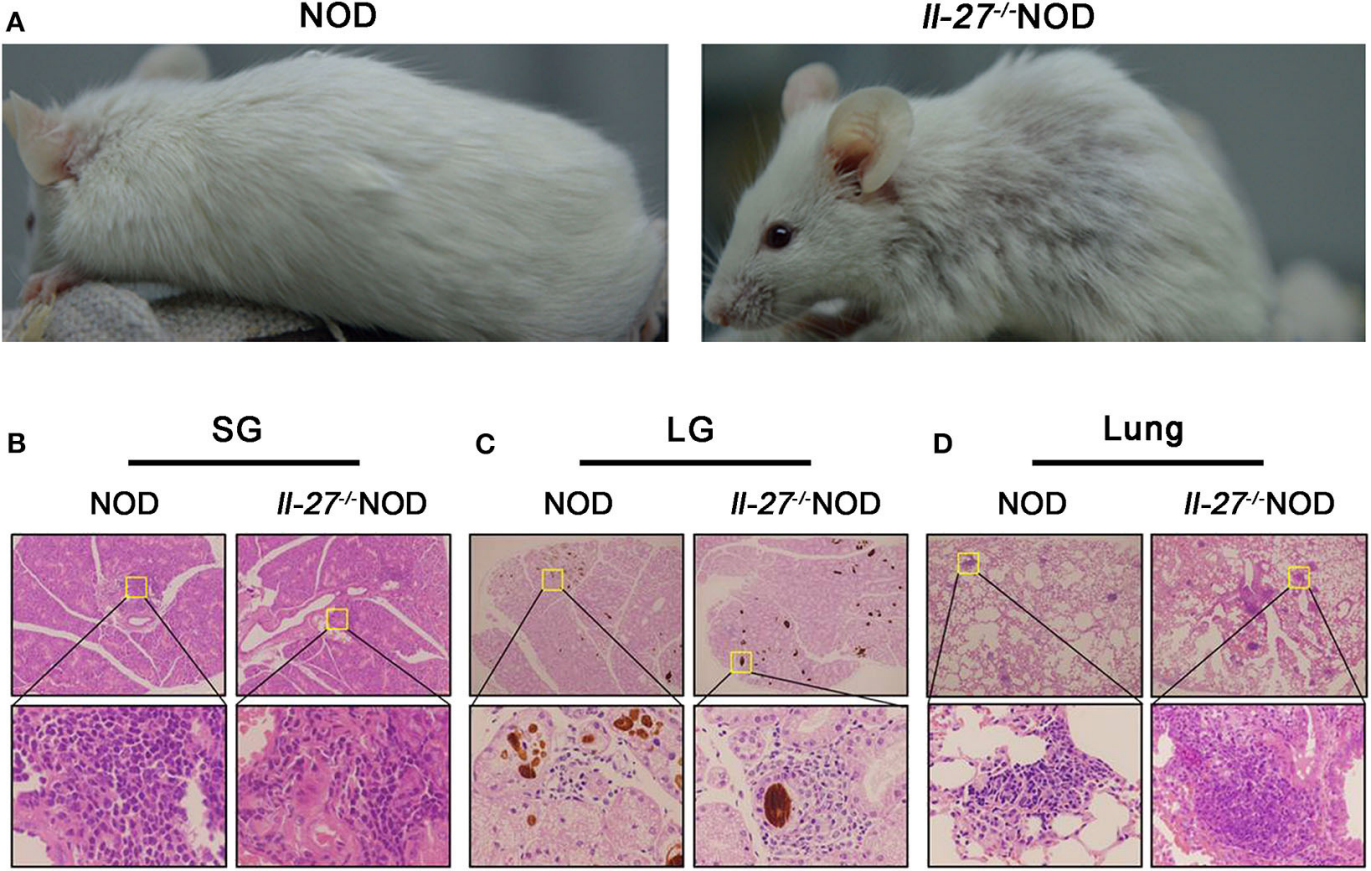
Interleukin 27 gene deficiency aggravated SS in NOD mice. **(A)** The physical appearance manifestation of rash in 12-week-old female NOD and *Il-27*^−/−^NOD mice. **(B–D)** Histological analysis, SGs, LGs, and lung from representative NOD and *Il-27*^−/−^NOD mice stained with hematoxylin and eosin to assess inflammation (top, magnification ×100; bottom, magnification ×400), *n* = 5.

**Figure 2:**
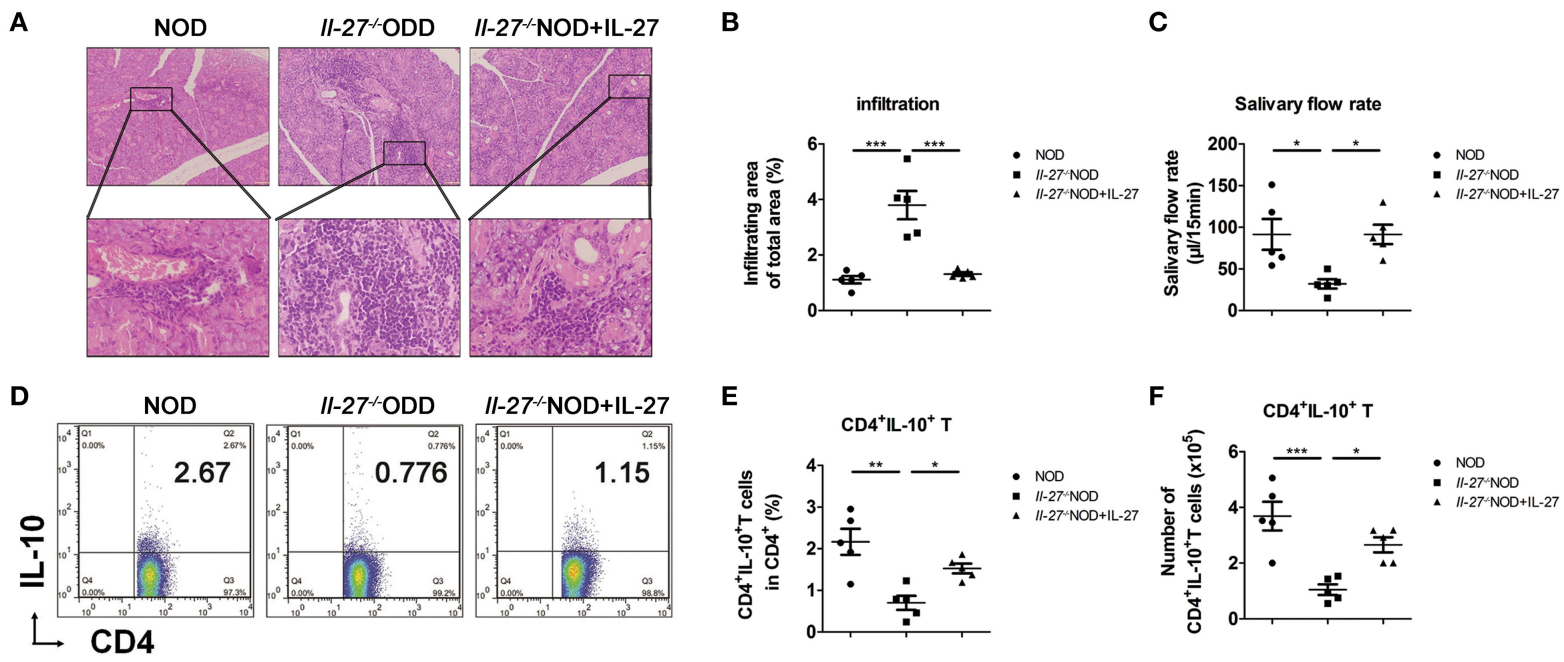
Lower levels of salivary flow rate and CD4^+^IL-10^+^ T cells in *Il-27*^−/−^NOD mice. **(A)** Histological analysis, SG from representative 12-week-old female NOD and *Il-27*^−/−^NOD and IL-27–treated *Il-27*^−/−^NOD mice stained with hematoxylin and eosin to assess inflammation (top, magnification ×100; bottom, magnification ×400). **(B)** The lymphocyte infiltration in SG of mice were evaluated for histological scores. **(C)** Salivary flow rate, **(D)** representative flow cytometry results, and **(E)** the percentage and **(F)** absolute number of splenic CD4^+^IL-10^+^ T cells in NOD and *Il-27*^−/−^NOD and IL-27–treated *Il-27*^−/−^NOD mice. Error bars indicate SEM. *p < 0.05, **p < 0.01, ***p < 0.001, *n* = 5.

Interleukin 27 has been shown to attenuate multiple autoimmune disorders by regulating the response of T cells and decreasing the production of inflammatory cytokines (34–36). To investigate the potential role of IL-27 in SS, we compared the severity of SS-like symptoms in *Il-27*^−/−^NOD and wild-type NOD mice. We found that *Il-27*^−/−^NOD mice developed rash (Figure 1A) and had swollen SGs compared to NOD mice (Figures S1A,B). Histopathologic analysis results showed more severe inflammation in SG (Figure 1B and Figure S1C), LGs (Figure 1C and Figure S1D), and lung (Figure 1D and Figure S1E) of *Il-27*^−/−^NOD mice. The level of salivary flow rate decreased significantly in *Il-27*^−/−^NOD mice (Figure 2C). These data indicated that the IL-27 deficiency aggravated SS in NOD mice.

### CD4^+^IL-10^+^ T Cells Decreased in *Il-27^−/−^*NOD Mice

To explore the effects of IL-27 gene deficiency on inflammation in NOD mice, the mice were divided into three groups: NOD, *Il-27*^−/−^NOD, and *Il-27*^−/−^NOD mice with IL-27 treatment. More infiltrating lymphocytes and larger infiltrating area were seen in SG of *Il-27*^−/−^ NOD mice compared to those of NOD and *Il-27*^−/−^ NOD mice treated with IL-27 (Figures 2A,B). We collected the whole saliva from the oral cavity and found the saliva volume of *Il-27*^−/−^ NOD mice reduced significantly, while exogenous IL-27 treatment could restore the salivary flow rate of *Il-27*^−/−^NOD mice (Figure 2C). The proportion and absolute number of splenic CD4^+^IL-10^+^ T cells of *Il-27*^−/−^NOD mice were lower than those of wild-type NOD mice. Exogenous IL-27 treatment restored the proportion (Figures 2D,E) and absolute number of splenic CD4^+^IL-10^+^ T cells (Figure 2F) significantly in *Il-27*^−/−^NOD mice. These findings suggested that IL-27 gene deficiency aggravated SS-like symptoms in NOD mice, and exogenous IL-27 treatment could reverse the decreasing salivary flow rate, splenic CD4^+^IL-10^+^ T cells, and serious inflammation.

### Exogenous IL-27 Up-Regulated CD4^+^IL-10^+^ T Cells in NOD Mice

**Figure 3:**
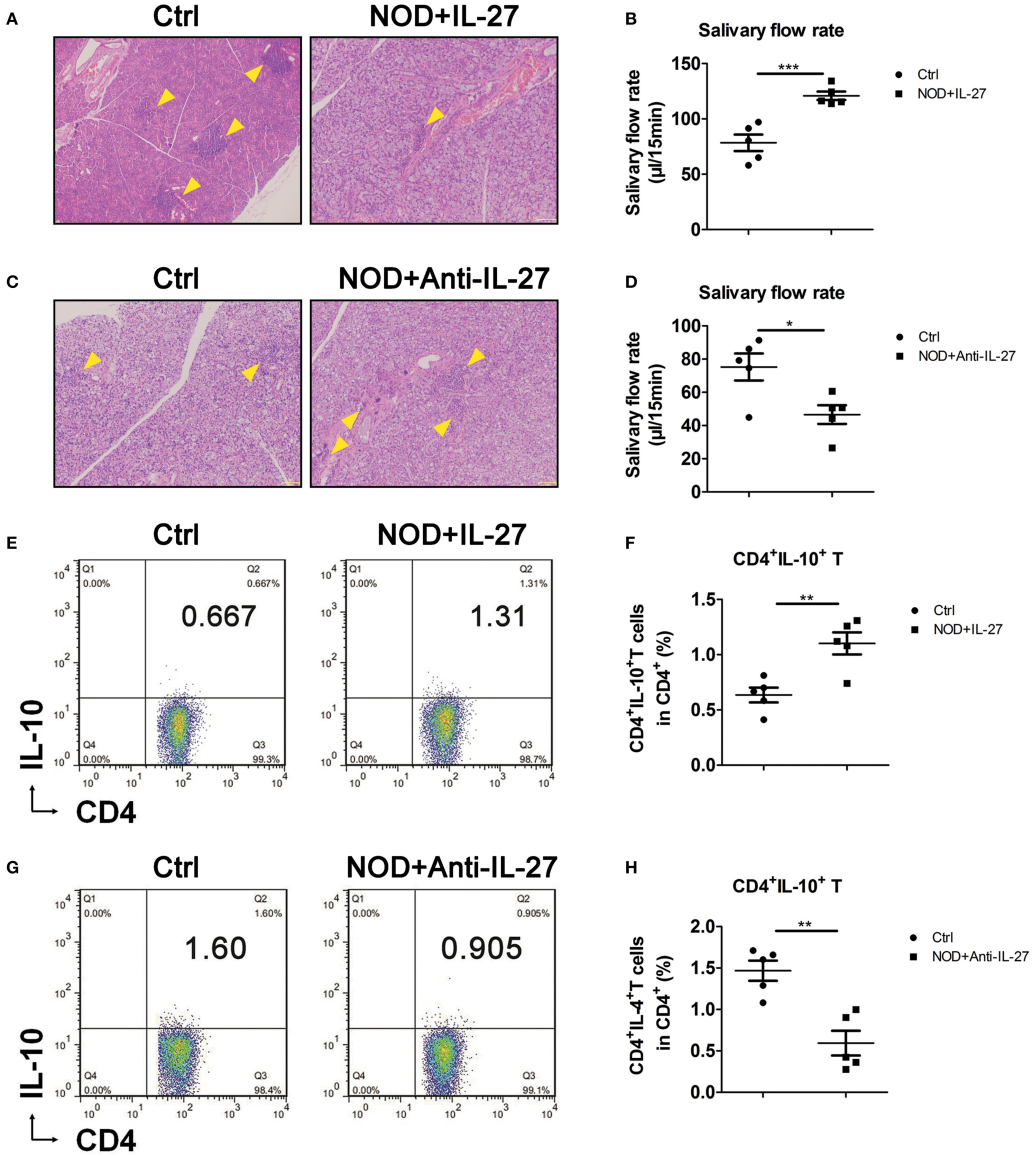
Interleukin 27 suppressed the inflammation in NOD mice. **(A,C)** Histological analysis, SG from representative mice stained with hematoxylin and eosin to assess inflammation (magnification ×100). Arrows indicate lymphocytes infiltrating focus. **(B,D)** Salivary flow rate of 12-week-old female NOD mice. **(E,G)** Representative flow cytometry results and **(F,H)** the percentage of splenic CD4^+^IL-10^+^ T cells in NOD mice. Error bars indicate SEM. *p < 0.05, **p < 0.01, ***p < 0.001, *n* = 5.

Given the aggravated SS-like symptoms and the decreased CD4^+^IL-10^+^ T cells in *Il-27*^−/−^NOD mice, we sought to investigate whether exogenous IL-27 treatment could up-regulate CD4^+^IL-10^+^ T cells and suppress inflammation in NOD mice. NOD mice were injected i.p. with recombinant IL-27, and the infiltration in SG, salivary flow rate, and splenic CD4^+^IL-10^+^ T cells were examined. We found NOD mice treated with IL-27 showed fewer lymphocytes in SG (Figure 3A) and LG (Figure S2A) and higher level of salivary flow rate (Figure 3B). Meanwhile, we used flow cytometry as the gating strategy (Figure S2C) to characterize the proportions and numbers of splenic CD4^+^IL-10^+^ T cells. We found significantly higher percentage, but not more numbers (Figure S2D), of splenic CD4^+^IL-10^+^ T cells in IL-27–treated NOD mice (Figures 3E,F). To further determine the significance of IL-27–induced of CD4^+^IL-10^+^ T cells in SS, anti-IL-27 and homologous isotype IgG2a were injected i.p. to NOD mice. As expected, we found the infiltration in SG (Figure 3C) and LG (Figure S2B) aggravated, the salivary flow rate reduced significantly (Figure 3D), and splenic CD4^+^IL-10^+^ T-cell proportion, but not the numbers (Figure S2E), decreased significantly (Figures 3G,H) in anti-IL-27–injected NOD mice. These results consist with our findings in *Il-27*^−/−^NOD mice and support the notion that IL-27 could inhibit SS-like symptoms in NOD by promoting CD4^+^IL-10^+^ T cells.

### IL-27 Promoted the Development of CD4^+^IL-10^+^ T Cells

**Figure 4:**
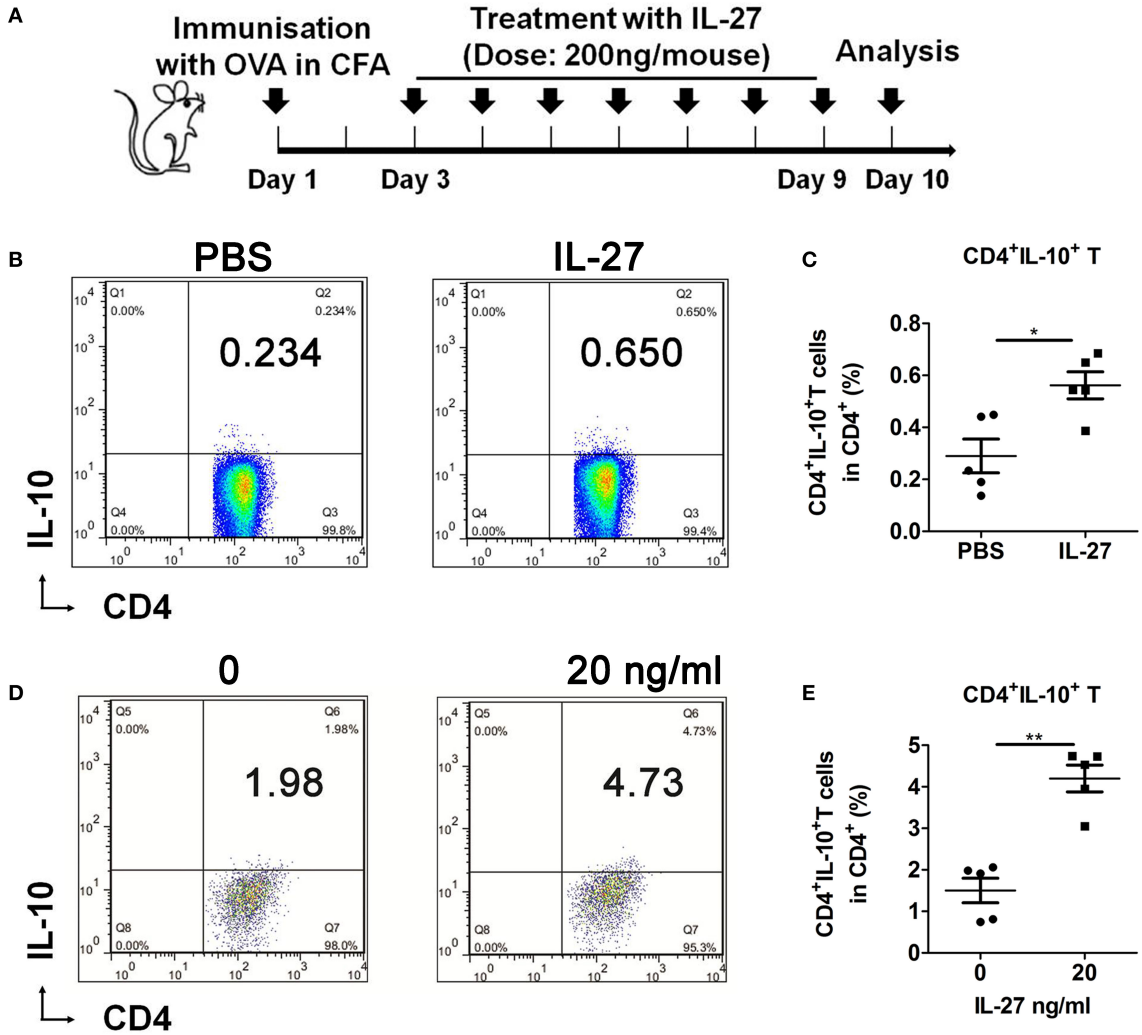
CD4^+^IL-10^+^ T cells generation and differentiation were promoted by IL-27. **(A)** IL-27 treatment schedule. **(B)** Representative flow cytometry results and **(C)** the percentage of splenic CD4^+^IL-10^+^ T cells in IL-27–treated or control OVA-immunized 9-week-old female C57BL/6 mice. **(D)** Representative flow cytometry results, and **(E)** the percentage of differentiated CD4^+^IL-10^+^ T cells. Error bars indicate SEM. *p < 0.05, **p < 0.01, *n* = 5.

Next, the C57BL/6 mice were immunized with OVA and treated with PBS or IL-27 as schedule (Figure 4A). Results showed that IL-27 remarkably promoted the generation of CD4^+^IL-10^+^ T cells *in vivo* (Figures 4B,C). In addition, we cultured splenic naive T cells from C57BL/6 mice with rIL-27 *in vitro* to investigate the effects of IL-27 on the differentiation of CD4^+^IL-10^+^ T cells *in vitro*. And we found that IL-27 up-regulated the differentiation of CD4^+^IL-10^+^ T cells significantly (Figures 4D,E). Collectively, these results suggested that IL-27 significantly promoted generation and differentiation of CD4^+^IL-10^+^ T cells *in vivo* and *in vitro*.

### IL-27 Correlated With CD4^+^IL-10^+^ T Cells in SS Patients

**Figure 5:**
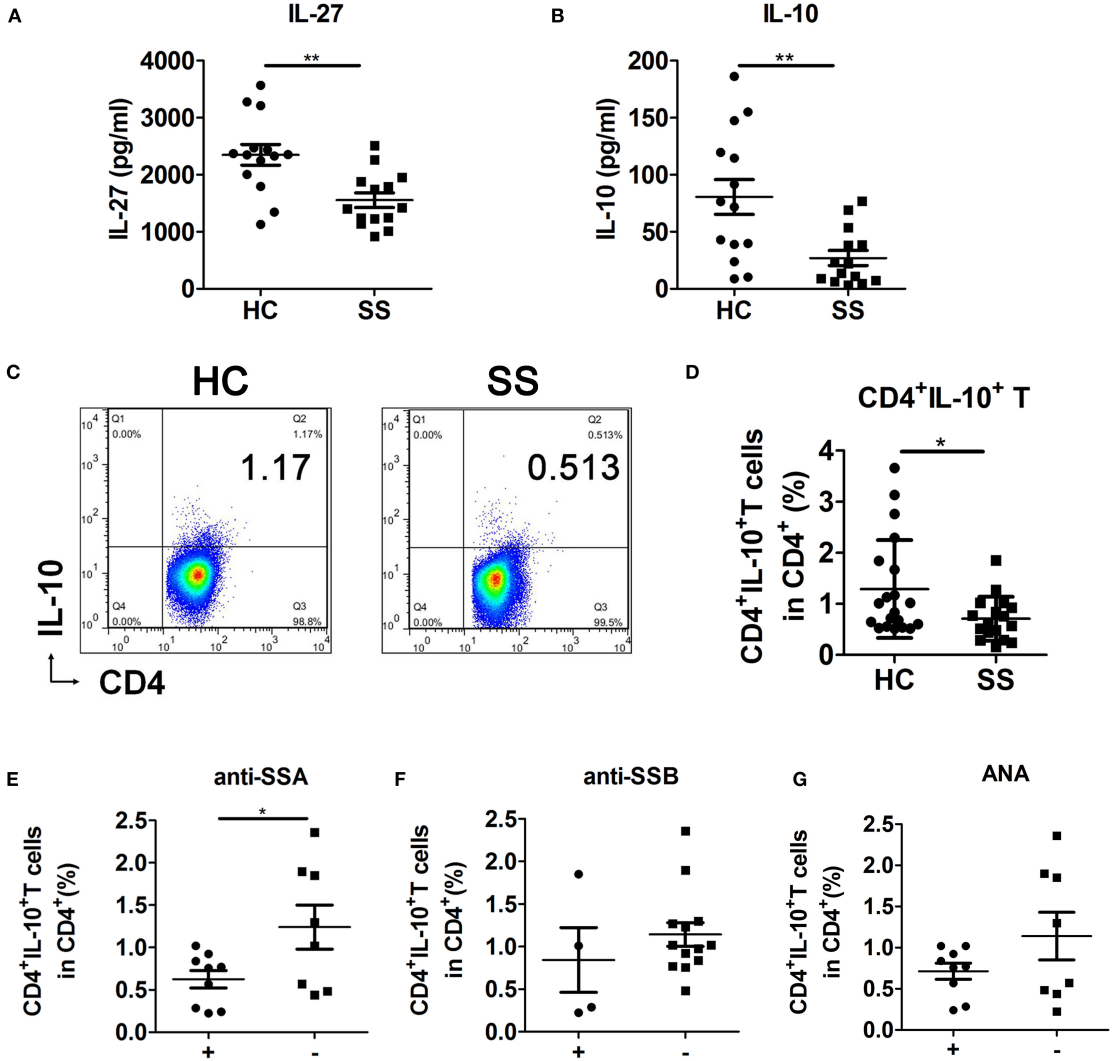
Interleukin 27 was related to decreased CD4^+^IL-10^+^ T cells in SS patients. **(A,B)** Plasma levels of IL-27 and IL-10 in HCs (*n* = 14) and SS patients (*n* = 14) were detected by ELISA. **(C)** Representative flow cytometry results and **(D)** the percentage of CD4^+^IL-10^+^ T cells in HCs (*n* = 20) and SS patients (*n* = 17). Sjögren syndrome patients were divided into antibodies-positive (+) and antibodies-negative (–) two groups. The correlations of CD4^+^IL-10^+^ T cells with **(E)** anti-SSA antibodies, **(F)** anti-SSB antibodies, and **(G)** ANAs were analyzed in SS patients. Error bars indicate SEM. *p < 0.05, **p < 0.01.

To examine the role and relationship of IL-27 and CD4^+^IL-10^+^ T cells in SS pathogenesis and whether they are correlated with known markers of SS disease, such as anti-SSA/Ro antibodies, anti-SSB/La antibodies, and antinuclear antibodies (ANAs). We detected the levels of plasma IL-27, IL-10, and peripheral CD4^+^IL-10^+^ T cells in HCs and SS patients. In addition, we analyzed the correlation between CD4^+^IL-10^+^ T cells and anti-SSA antibodies, anti-SSB antibodies, and ANAs in SS patients (Table S1). Results showed that the levels of plasma IL-27 (Figure 5A) and IL-10 (Figure 5B) and percentage of peripheral CD4^+^IL-10^+^ T cells (Figures 5C,D) in SS patients were lower than those in HCs. CD4^+^IL-10^+^ T cells were negatively correlated with anti-SSA antibodies (Figure 5E), but not anti-SSB antibodies (Figure 5F) and ANAs (Figure 5G). These data support the view that CD4^+^IL-10^+^ T cells regulated by IL-27 participated in SS pathogenesis.

## Discussion

Our study here has shown that IL-27–regulated CD4^+^IL-10^+^ T cells participated in SS pathogenesis. *Il-27*^−/−^NOD mice displayed severe SS disease. Exogenous IL-27 improved the symptoms of SS by promoting the generation of Tr1 cells in *Il-27*^−/−^NOD and NOD mice. And, antibody neutralization of IL-27 not only exacerbated inflammation but also reduced splenic CD4^+^IL-10^+^ T cells significantly in NOD mice. Moreover, SS patients have lower levels of IL-27, IL-10, and CD4^+^IL-10^+^ T cells. And, CD4^+^IL-10^+^ T cells were negatively correlated with anti-SSA antibodies. Together, these results have demonstrated a critical role of IL-27 in the pathogenesis of SS.

The anti-inflammatory properties of IL-27 have been reported in several autoimmune diseases, and IL-27 has been proposed as a therapy to modify inflammatory conditions by regulating T-cell responses (32). However, the contradictory effects of IL-27 have been reported in type 1 diabetes (T1D). Recent investigation reported that IL-27 not only showed immunomodulatory function, but also was a compensatory effort of dendritic cells against the ongoing inflammation in T1D patients (37). On the contrary, Ciecko et al. (38) reported that IL-27 contributes to the pathogenesis of T1D in NOD mice by altering the balance of Treg and T_H_1 cells and enhancing the effector function of CD8 T cells, although it was reported that serum IL-27 was strongly elevated in patients with SS (39), which is inconsistent with our results. This may be because SS patients were particularly associated with interstitial lung disease complications in their study. Another study reported that the levels of IL-27 and IL-23 increased significantly in the serum and urine of systemic lupus erythematosus patients with and without lupus nephritis compared with healthy control (40), which may account for the pleiotopic roles of IL-27 in several cases of autoimmunity. Up to date, many studies have reported that IL-27 showed a protective role in a variety of diseases, and exogenous IL-27 could suppress multiple autoimmune diseases by promoting Tr1 cells or other mechanisms (6, 16, 35, 36, 41, 42). Lee et al. (43) reported that exogenous IL-27 could induce a suppressive effect on SS development by regulating T_H_17 pro-inflammatory activity. Our previous study showed that IL-27 decreased significantly in SS patients and SS-like NOD mice, and mesenchymal stem cells (MSCs) alleviated SS by elevating the level of IL-27 (44). In this study, we found that IL-27 alleviated SS by inducing IL-10–producing CD4^+^ T cells. The different properties of IL-27 in the pathogenic conditions may account for the complex effects of IL-27 on different lymphocyte populations, which play a pleiotropic role in the development and progression of disease in NOD mice. Thus, the specific effects of IL-27 in SS need further investigation. In our studies, we compared the SS-like symptoms in NOD and *Il-27*^−/−^NOD mice and found the protective function of IL-27 by regulating Tr1 cells in SS.

Tr1 cells, a T-cell population with distinct suppressive function, are characterized by secreting high amounts of IL-10 and variable amounts of IFN-γ, IL-2, and TGF-β, depending on the microenvironment and the disease context (23), while none of the biomarkers are yet the ideal candidates for Tr1 cells. Studies suggested that IL-10–producing CD4^+^ T cells may represent a heterogeneous cell population reflecting different cell origins, maturation stage, or different functions of Tr1 cells (45). Tr1 cells suppressed immune responses mainly by producing IL-10 (23). A number of investigators have also documented that Tr1 cells could prevent T cell–mediated diseases via a TGF-β-dependent mechanism in addition to IL-10 (46, 47). Interleukin 27 was the dominant factor for IL-10–producing T cells and worked together with TGF-β to further enhance Tr1 differentiation (23). And IL-27 limited autoimmune disorders by promoting Tr1 cells. In this study, exogenous IL-27 ameliorated SS-like syndromes in NOD mice by promoting the generation of CD4^+^IL-10^+^ T cells in NOD and *Il-27*^−/−^NOD mice, whereas IFN-γ and TGF-β as the important factors involved in the induction and function of Tr1 cells should be considered and need to be identified.

Although the deficiency of IL-27 signal resulted in more serious SS-like symptoms and fewer splenic CD4^+^IL-10^+^ T cells in NOD mice, the percentage of CD4^+^IL-10^+^ T cells was very low and showed no significant difference in SG of NOD and *Il-27*^−/−^NOD mice (Figure S3). The improved SS-like syndromes in IL-27–treated NOD mice might be due to that splenic CD4^+^IL-10^+^ T cells could affect already established inflammation in SG and saliva flow indirectly via IL-10 to regulate other immune responses in the immune system.

Many different cytokines can induce JAKs and STATs activation to regulate fundamental biological processes (48). It has been reported that IL-27 induced IL-10–producing Tr1 cells generation by activating STAT1 and STAT3. While other cytokines that signal, such as IFN-α or IL-6, alone or in combination, could promote the generation of Tr1 cells via STAT3 (49, 50). Brockmann et al. reported that IL-10 signaling controlled the differentiation and regulatory activity of Tr1 cells via P38MAPK and not by STAT3 (51). These studies suggested that STAT1 and/or STAT3 were activated in the Tr1 cells depending on different cytokine signals.

In summary, we have demonstrated that IL-27 could promote the development and differentiation of CD4^+^IL-10^+^ T cells *in vitro* and *in vivo*. Interleukin 27 gene deficiency resulted in decreased CD4^+^IL-10^+^ T cells and aggravated the severity of SS-like symptoms of NOD mice. Exogenous IL-27 ameliorated *Il-27*^−/−^NOD and NOD mice by regulating CD4^+^IL-10^+^ T cells. Notably, CD4^+^IL-10^+^ T cells decreased in SS patients, which may be correlated to IL-27. These findings suggested that IL-27 played a crucial role in the pathogenesis of SS by regulating CD4^+^IL-10^+^ T cells. We propose that targeting IL-27 and CD4^+^IL-10^+^ T cells may be a new direction for the SS treatment.

## Data Availability Statement

The raw data supporting the conclusions of this article will be made available by the authors, without undue reservation.

## Ethics Statement

The studies were reviewed and approved by Nanjing Drum Tower Hospital, The Affiliated Hospital of Nanjing University Medical School. Written informed consent was obtained from all subjects.

## Author Contributions

JQ and ZZ participated in study design, data collection, data analysis, data interpretation, and drafting the paper. XT, WL, and WC participated in patient recruitment, animal experiments, and data collection. GY supervised the whole research, designed the study, interpreted the data, and wrote the paper. All authors read and approved the manuscript.

## Conflict of Interest

The authors declare that the research was conducted in the absence of any commercial or financial relationships that could be construed as a potential conflict of interest.
