# Supplementary material for: IL-27 Regulated CD4+IL-10+ T Cells in Experimental Sjögren Syndrome
Source: Front Immunol. 2020 Aug 11;11:1699. doi: 10.3389/fimmu.2020.01699 (PMC7431603; doi:10.3389/fimmu.2020.01699)
Supplement: Supplementary file 1 [file Data_Sheet_1.PDF]

SUPPLEMENTARY FIGURE LEGENDS

FIGURE S1. Representative images**(A)**and analysisof submandibular gland weight**(B)**ofNOD and *Il-27*$^{-/-}$NOD mice.Histological analysisof SG**(C)**, LG**(D)** and Lung**(E)**from NOD and *Il-27*$^{-/-}$NOD mice. Error bars indicate SEM. *, $p<0.05$, **, $p<0.01$, ***, $p<0.001$, n=5.

FIGURE S2.(A) Representative histological images(400x).LGfrom representative control (NOD mice treated with PBS) and NOD mice treated with IL-27stained with hematoxylin and eosin to assess inflammation.**(B)** Representative histological images(400x). LGfrom representative control (NOD mice treated with IgG2a) and NOD mice treated with anti-IL-27.**(C)**Gating strategy used to identify CD4+IL-10+ T cells. In the morphological gate (FSC/SSC) we excludeddebris, then we gated CD4+ T cells and CD4+IL-10+ T cells were selected. **(D and E)** The absolute number of splenic CD4+IL-10+ T cells in IL-27 oranti-IL-27treatedNOD mice and the control mice, respectively.

FIGURE S3. Representative flow cytometry results of CD4+IL-10+ T cells in SG of NOD and *Il-27*$^{-/-}$NOD mice.

Figure S1

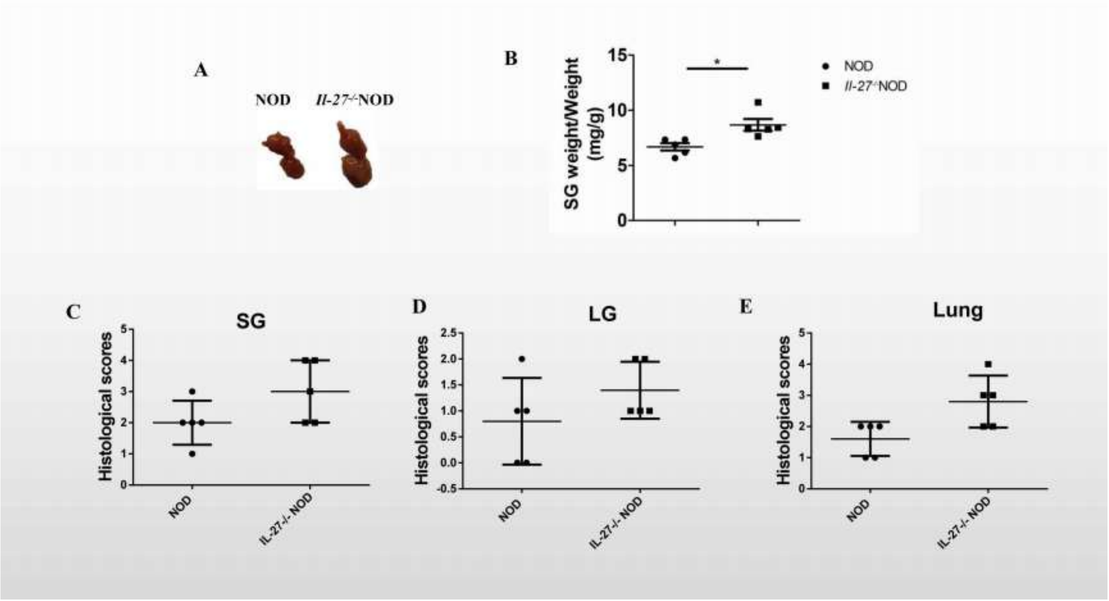

A
NOD
Il-27-/-NOD
B
SG weight/Weight (mg/g)
*
NOD
Il-27-/-NOD
C
SG
Histological scores
NOD
IL-27-/- NOD
D
LG
Histological scores
NOD
IL-27-/- NOD
E
Lung
Histological scores
NOD
IL-27-/- NOD

Figure S2

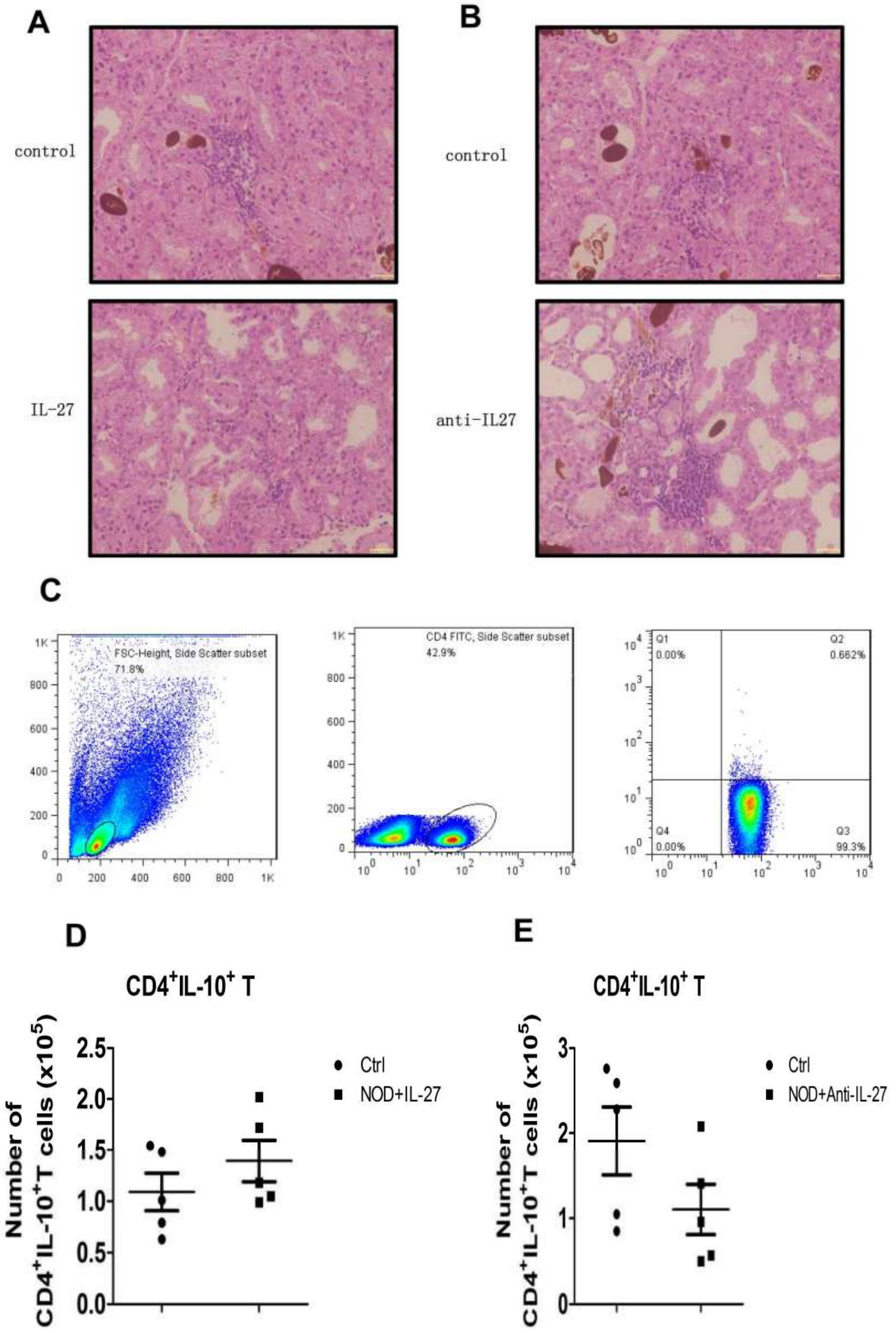
A
control
IL-27
B
control
anti-IL27
C
FSC-Height, Side Scatter subset
71.8%
CD4 FITC, Side Scatter subset
42.9%
Q1
0.00%
Q2
0.662%
Q4
0.00%
Q3
99.3%
D
CD4+IL-10+ T
Number of CD4+IL-10+T cells (x10^5)
Ctrl
NOD+IL-27
E
CD4+IL-10+ T
Number of CD4+IL-10+T cells (x10^5)
Ctrl
NOD+Anti-IL-27

Figure S3

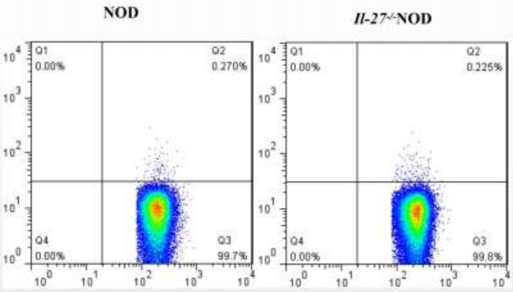

CD4+IL-10+ T cells in SG

Supplementary Table 1 Clinical characteristics of the SS patients

**SS Patient**	
**n**	31
**Age**	48.1±10.2
**sex**	M/F 3/28
**Disease duration (months)**	1-240(median 50.3)
**ESSDAI**	6.3±2.1
**Anti-SSA % n**	61.3%(19)
**Anti-SSB % n**	12.9% (4)
**ESR (mm/h)**	29.32±4.23
**CRP(mg/dL)**	2.02±0.61
**IgG (g/L)**	14.13±2.32

ESSDAI, EULAR 2010 Sjögren's syndrome disease activity index, ESR,erythrocyte sedimentation rate, CRP, C-reactive protein
